# Postnatal auditory preferences in piglets differ according to maternal emotional experience with the same sounds during gestation

**DOI:** 10.1038/srep37238

**Published:** 2016-11-18

**Authors:** Céline Tallet, Marine Rakotomahandry, Carole Guérin, Alban Lemasson, Martine Hausberger

**Affiliations:** 1 I.N.R.A., U.M.R.1348 PEGASE, Saint-Gilles, France; 2 Agrocampus Rennes, U.M.R.1348 PEGASE, Rennes, France; 3 Université de Rennes 1, Laboratoire d’éthologie animale et humaine, U.M.R. 6552- C.N.R.S., Paimpont, France; 4 C.N.R.S., Laboratoire d’éthologie animale et humaine, U.M.R. 6552- Université de Rennes 1, Rennes, France

## Abstract

Prenatal sensory experience, notably auditory experience, is a source of fetal memories in many species. The contiguity between sensory stimuli and maternal emotional reactions provides opportunity for associative learning *in utero* but no clear evidence for this associative learning has been presented to date. Understanding this phenomenon would advance our knowledge of fetal sensory learning capacities. In the present study we tested the hypothesis that sounds (human voice) broadcast to pregnant sows while they experienced positive or negative emotional situations influences postnatal reactions of their offspring to these same sounds. The results show that: 1) from the first testing at the age of 2 days, the experimental piglets were less distressed by a social separation than controls if they heard the “familiar” voice, 2) piglets generalized to any human voice although the influence of novel voices was less pronounced, 3) in a challenging situation, piglets were more distressed if they heard the voice that was associated with maternal negative emotional state *in utero*. These findings open a whole line of new research on the long term effect of *in utero* associative learning that goes well beyond pigs, providing a framework for reconsidering the importance of sensory and emotional experiences during gestation.

Fetuses of many species are able to perceive external sensory stimuli, such as sounds, at least in the last phase of their development^1^. In birds, crocodiles and mammals the perception of acoustic stimuli has been demonstrated through neural^2^,^3^, behavioural^4^,^5^,^6^,^7^ and physiological^4^,^7^,^8^ responses. Fetuses perceive both species-specific stimuli like maternal^4^ or unfamiliar voices^8^, and non-specific ones like music^2^ or pure tones^3^. Human fetuses express discriminative responses to prenatally heard stimuli compared to unfamiliar ones: e.g. musical notes^9^ and maternal voice^10^. This prenatal experience of a melody modulates the brain’s event-related potentials till at least 4 months of age in humans^11^. Human and sheep neonates express habituation to stimuli repeatedly heard prenatally^12^,^13^, demonstrating transnatal transmission. In pigs, birds and humans, neonates may show preferences for sounds in their prenatal environment early after birth^14^,^15^,^16^ which may influence the development of social bonds^17^ and vocal production^18^. Human neonates are able to discriminate their father’s voice compared to a male voice but do not show voice preferences^19^,^20^, suggesting that prenatal experience would have distinctive effects according to the stimulus quality.

Transnatal transmission has been demonstrated for different modalities: in rats, prenatal ethanol exposure correlates with postnatal affinity to the drug^21^; fetal conditioning with chemical stimuli is maintained after birth^22^,^23^; pregnant females’ exposure to space flight induces changes in the vestibular characteristics of newborn pups^24^. Maternal movements during pregnancy seem to have a durable effect on fetuses. Fetuses in a variety of species are able to transduce sensory information (rats, cats, lambs)^25^, but maternal behavior and physiology is considered as a major source of fetal sensory experience^25^. It is well known that maternal emotions can have a lasting influence on offspring’s reactivity from an early postnatal age and may condition the associations they form with environmental stimuli^26^,^27^. But this influence also occurs at much earlier stages, such as the fetal stage. It has been demonstrated in humans that the emotional state of the pregnant female during experimental tasks is reflected in changes in heart rate and heart rate variability of the fetus: it seems that variations in women’s emotion based physiological activity can affect the fetus^28^,^29^, especially if they are scored as being anxious^28^,^30^.

Despite the fact that there is associative learning as well as sensitivity to maternal emotions at the fetal stage, to our knowledge no study has investigated the impact of maternal emotional state on the fetal memory of sensory stimuli and its transnatal potential transmission. The aim of the present study was to test the hypothesis, using an animal model, that sounds broadcast during gestation, while the sow experiences a negative or positive situation, may have an impact on the postnatal reactions of their offspring to these sounds. Mastropieri & Turkewitz^31^ have shown that newborns are capable of interpreting the vocal expression of emotions and hypothesized that contiguity between the acoustic stimuli and the maternal reaction “would provide opportunity for associative learning (via classical conditioning) in utero”^31^ (see also Parncutt^32^). However, the potential role of maternal emotions in guiding sensory learning in fetuses is poorly known. One can wonder, for instance, if the postnatal preferences of human newborns for (prenatal) familiar music may develop as a consequence of maternal positive states experienced while listening. Associating the internal emotional reactions to particular sounds has an obvious adaptive value for neonates; for example, they could react appropriately if hearing a sound indicating danger.

In the present study we used an animal model, the pig, to test this hypothesis. Thirty pregnant sows were submitted, during their last month of pregnancy, to both positive (e.g. soft brushing) and negative (e.g. electric prod) handling^33^,^34^,^35^,^36^ (10 minutes each five days per week, alternating the type of treatment morning and afternoon every day) while hearing (V) or not (C) a human voice. In order to avoid any conclusion related to the particular acoustics of a single voice, one group (V1+) was positively handled by experimenter 1 while hearing voice 1 and negatively by experimenter 2 while hearing voice 2, and vice versa for V2+ (Fig. 1). Previously recorded voices were broadcast through a loudspeaker that was worn by the experimenter during the handling. The recordings consisted of reading a standardized text repeated continuously during the handling procedure. No human voice was heard by the animals outside the experimental sessions at any stage (from early pregnancy until the end of the tests on the piglets at 21 days postpartum; caretakers were informed). For each experimental group therefore, each voice was associated with a particular valence of handling: V1 with a positive valence for V1+ and negative valence for V2+.

The purpose of this experiment was to determine if piglets are capable of auditory associative learning while in the womb that persists to the postnatal period. The experiment included both tests for valence specific associations and generalization effects. Valence specific associations were tested via behavioral responses to a unique voice associated with either a positive or a negative emotional experience for the sow *in utero*; and generalization effects were tested by assessing piglets’ responses to novel human voices after conditioning.

In order to test whether the broadcast of voices to the sows during gestation had an impact on the behavior of piglets after birth, piglets were submitted, after birth, to a series of classical standardized tests, starting at 2 days of age (Table 1) and aiming at evaluating three aspects (note that the situation number corresponds to the chronology and not to order of research questions):

1. Is there a postnatal memory of prenatally broadcast sounds? If the human voice had become familiar through prenatal broadcast, we expected it to reduce the effects of an unfamiliar and stressful situation such as social isolation^33^,^37^. Therefore the piglets were tested in tests of social isolation on postnatal days 2, 7 and 21. During Social Isolation Test 1 (SIT1, 2 days old), the piglet was alone in an unfamiliar corridor pen while the two prenatally broadcasted voices (V1 + V2, because both were familiar independently of valence) were played back. During Social Isolation Test 2 (SIT2, 7 days old), the piglet was alone in an unfamiliar square area, first with no sound broadcast (Phase 1) and then with one voice (V1 or V2) broadcast (Phase 2). During Social Isolation Test 4 (SIT4, 21 days old), the piglet was moved again to the square area but with a motionless and silent unfamiliar human^33^ and with the playback of the voice prenatally associated with maternal positive treatment (i.e. V1 for V1+ and V2 for V2+). In all situations, the behaviors of experimental (V) and control (C) piglets when being exposed to human voices (familiar for V and unfamiliar for C) were compared.
2. Is there generalization of one type of prenatal external auditory stimulation? Here we tested whether piglets whose mothers were submitted to the playback of human voices during gestation reacted differently from controls when a novel human voice was broadcast. Thus, the piglets were submitted to the broadcast of two novel human voices on postnatal day 14 (V3 + V4, both unfamiliar independently of any potential emotional valence, reading the same text). This Social Isolation Test 3 (SIT3) was identical to SIT1 apart from the voice identities. Again, the behaviors of V and C piglets were compared.
3. Is there transnatal transmission of the emotional valence associated with a particular sound from the pregnant sow to the fetus? Here, we compared how experimental piglets (V) behaved when hearing the voice prenatally associated to maternal positive treatment (i.e. V1 for V1+ and V2 for V2+) and the voice prenatally associated to maternal negative treatment (i.e. V2 for V1+ and V1 for V2+) compared to o a situation where no sound was broadcast. Hence, phases 1 and 2 of SIT2 were compared.

For all Social Isolation Tests, we quantified the number of distress vocalizations (i.e. so-called high pitched distress calls^38^,^39^) emitted by piglets. This is a measure commonly used to assess the level of stress experienced by pigs^40^. Outside the experiments and until their conclusion, no handling or voice stimulation was given to either the sows or the piglets.

## Results

Data for sows were analyzed using nonparametric statistics. For the piglets, we used mixed models. In a first step, all meaningful factors were included as fixed factors in the model (i.e. according to the experimental test: group, voice, voice emotional valence, phase - for SIT2 only). As there was no significant voice effect in any model tested (p > 0.05), data of V1+ and V2+ piglets were pooled and are thus now called group V. This revealed that the effects of auditory experience did not depend on mere voice quality.

### Reactions of the sows to the handling procedure

Behavioural reactions of the pregnant females during the handling sessions confirmed the respective valence of the negative and positive handlings. Thus, during handling sessions, all sows expressed more distress calls (19% of observations (10–40) *versus* 0 (0–1); Wilcoxon = 465, N = 28, p < 0.0001), more withdrawal (42% of observations (35–53) *versus* 1 (0–5); Wilcoxon = 465, N = 28, p < 0.0001), more postural changes (5% of observations (2–7) *versus* 1 (0–2); Wilcoxon = 456, N = 28, p < 0.0001), and more aggressiveness (number of animals expressing aggressiveness at least once: Npos = 4 out of 28 sows, Nneg = 13 out of 28; Fisher exact test, p = 0.02) during the negative than during the positive sessions. Sows reacted similarly to the procedures whether or not voice was broadcast (Nc = 10, Nv = 20, U = 331, p > 0.28). When only the control animals (N = 10) were considered, the same differences according to treatment appeared (Distress calls: Wilcoxon = 55, p = 0.002; Withdrawal: Wilcoxon = 55, p = 0.002; Postural changes: Wilcoxon = 55, p = 0.002). But, interestingly, one difference could be observed between the control and experimental sows: the experimental sows were silent more often than the control sows during the sessions (66% of the sessions (49–68) *versus* 42% (33–52); U = 265, p = 0.03) as if “paying attention” to the auditory stimuli^41^.

### Testing a potential postnatal memory of prenatal auditory stimulation

At 2 days of age (SIT1), the piglets were tested individually while the two voices (V1 and V2) were broadcast. All piglets produced distress calls typical of the responses to this social separation^38^. However, the experimental piglets produced fewer distress calls when hearing the prenatally broadcast voices than the control piglets (F_1/26_ = 5.51, p = 0.03, Fig. 2a). Similar results were obtained at 7 days of age (SIT2 – Phase 2), when piglets were tested individually in an unfamiliar pen (F_1/26_ = 11.5, p = 0.002, Table 1): experimental piglets produced less distress calls when hearing the prenatally broadcast voices than the control piglets. Finally, similar results were obtained at 21 days of age (SIT4), when piglets were tested individually with an unfamiliar motionless human while one voice was broadcast (V1 for V1+ and V2 for V2+): experimental piglets produced fewer distress calls when hearing the prenatally broadcast voices than the control piglets (V: 62 ± 29 distress calls, C: 170 ± 43; F_1/24_ = 4.29, p = 0.49). This suggests that there was memory for the sounds heard prenatally and, hence, the sounds were more familiar and potentially reassuring after birth.

### Testing potential generalization of prenatal auditory memory

Interestingly, these effects of prenatal experience with a human voice seemed to generalize to new exemplars as the experimental (V) piglets, at 14 days of age (SIT3) also produced fewer distress calls than the control piglets (C) when hearing novel human voices (V3 and V4) (not heard by any of the sows or piglets) when socially isolated (Table 1) (F_1/13_ = 10.2, p = 0.007, Fig. 2b). The lowered response to voices generalized to unfamiliar – but still human – voices, suggesting a generalization of learning.

### Is there transnatal transmission of the emotional valence associated with a particular sound from the pregnant sow to the fetus?

For this question, only the prenatally stimulated piglets (V groups) were tested on postnatal day 7. They showed a clear influence of prenatal maternal experience related to the voices (Fig. 3). In this second social isolation test (SIT2), V piglets emitted more distress calls when the negative valence voice (phase 2) was played back than when no sound (phase 1) was played back (F_1/36_ = 4.82, p = 0.03). However, there was no effect of broadcasting the positive valence voice (phase 2) compared to no sound (phase 1) (F_1/35_ = 1.70, p = 0.20).

## Discussion

This study, based on the broadcast of human voices while pregnant sows were handled, reveals that prenatal audition of such sounds influences postnatal reactions of piglets towards them: their broadcast during a stressful situation (social separation) induces lowered stress reactions. Moreover, this impact of prenatal experience seems to induce a general “familiarity” with human voices as this reaction is also observed when novel unfamiliar human voices are broadcast to the piglets. Although one could argue that voices had acted as distractors, reducing the piglets’ vocal production, the fact that only the experienced piglets did so indicates that the decreased vocalization in the treatment groups was influenced by their experience with human voices during gestation. More interesting still is the finding that there is transnatal transmission of the negative emotional valence associated with the voice playback. Thus, after birth, the piglets reacted to social separation with more distress calls if they heard the voice that had been broadcast to their mother during gestation while they were experiencing a negative emotional situation than if they were kept in silence (or with the voice associated in utero with maternal positive experience). Maternal emotional reactions, therefore, are one channel of learning for fetuses. This supports the hypothesis of classical conditioning to maternal reactions^31^,^32^. In addition to being a potential explanation of how fetuses learn to recognize vocal expression of emotions, similar processes might be involved in the observation that music may acquire appeasing properties in human babies.

Although it can be argued that the repeated tests led to a familiarization of the piglets to the sounds, the finding that their behavior was influenced by the prenatal auditory experience at the very first testing at the age of 2 days shows that there was indeed a transnatal transmission and that it may have formed the basis for future reactions to these same stimuli but also to novel exemplars. The use of a non-species-specific stimulus enabled us to control the prenatal auditory experience but also to reveal that piglets are able, from this early learning, to generalize to other related sounds. In fact they seemed to be capable of both generalization and discrimination between voices. One limitation of the study is the absence of a reverse control condition. The present study constitutes an important baseline for future simpler designs associating behavioural and physiological responses and testing a variety of auditory stimuli (noise, music, other animal calls…) that may occur in the environment.

The most striking finding from the present study is perhaps the fact that piglets, during the postnatal period, were more disturbed by the social isolation if it was associated with the voice heard prenatally while their mother encountered a negative emotional experience. This seems a highly adaptive process by which young may, at birth, be able to identify and avoid potentially noxious stimuli through their sensory characteristics. External sounds are clearly perceived by animal and human fetuses^1^,^2^,^3^,^4^,^5^,^6^,^7^ and acoustic associative memories are probably the easiest modality to deliver such information postnatally.

The mechanisms involved in the prenatal associative memory for auditory stimuli and emotional experience are not known but the stress (and potentially the anxiety state due to repetition of the procedure) experienced by the sow during the negative handling likely created hormonal, kinesthetic, and/or vestibular changes to the intrauterine environment^24^,^25^,^28^,^29^. Fetal heart changes would be a great way to begin to understand the interplay between the auditory stimuli and emotional experience, similar to what has been done in human fetuses^30^. Future studies on pigs and other species should help disentangle the various processes involved in the prenatal transmission of both positive and negative emotional experiences.

These novel findings open a whole line of new research that goes well beyond the pig model. Thus, they have practical implications in terms of advice for pregnant women^42^. Confirming the conditioning process through direct measures of the fetal reactions will be the next step in order to further validate these findings. Functional brain imaging is one promising tool for this^43^. In any case, our results provide a basis for further investigation of the cognitive abilities of fetuses and their ability to perform complex associative learning in order to build lasting memories, a highly adaptive process.

## Methods

The design of the experiment was approved by the local ethics committee (Comité Rennais d’Ethique en matière d’Expérimentation Animale, number R-2012-CT- 01). The methods were carried out in accordance with French rural and sea fishing code’s articles R.214-87 to R.214-126.

### Animal model

The study used domestic pigs as a model. Pigs are social animals that are good models of humans in many neurological, physiological and behavioral aspects^44^. Vocal signals are their main way of communicating^45^. Pigs express a large variety of sounds that depend on the situation. Negative states lead to the emission of high, long and tonal sounds that are frequency modulated (screams) or not (squeals) while positive states induce lower, shorter and more tonal calls (croaks)^39^. Thus vocal expression can be used to identify their emotional state quite accurately, and researchers typically distinguish high-pitched stress vocalizations from low-pitched neutral or positive vocalizations^46^. In addition piglets express preferences for specific maternal acoustic signals from birth^17^, suggesting an important role of prenatal experience. Domestic pigs are familiar with humans, and thus the human voice was used as vocal signal and human contact served to induce positive or negative emotional states.

### Human voices

Vocal signals consisted of the experimenters E1 and E2 reading a text broadcast through loudspeakers (Mipro MA-100su, Mipro Electronics Co, Taiwan) at 90 dB measured at 1 m distance. The text contained all the phonemes of the French language, and had no emotional connotation: “*Petit Louis, les yeux ouverts, rêvait dans son lit bleu. Le jour des vacances était arrivé. Il sentait l’odeur du bon pain chaud et du chocolat que maman préparait. Papa et lui iraient à la gare chercher son cousin. Ils feraient du camping à la campagne. Louis n’aurait plus peur des ruades de l’âne brun*.” The text lasted 17 s and was repeated 35 times during the 10-min handling sessions. Voices were recorded with a professional microphone Sennheiser MKH50 P48 (Sennheiser, Germany) connected to a Marantz PMD661 recorder (Marantz Europe, The Netherlands). Two other experimenters (E3 and E4), unfamiliar to the piglets, were also recorded reading the same text.

### Sow handling treatments

**Figure 1:**
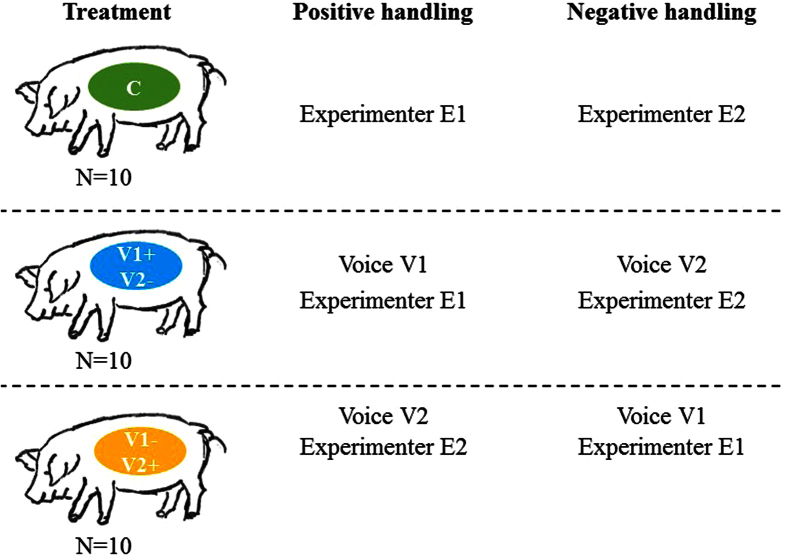
Schematic representation of experimental treatments to the sows: C = **control received the handling sessions but no voice.** V1+ and V2+ = experimental groups which received both handling and the broadcast of a human voice reading the text through a loudspeaker. In order to make sure the results were not just due to one voice quality, two experimenters (E1 and E2) performed the handling, wearing the loudspeaker (in order to have standardized acoustic parameters) that broadcast their own voice. We acknowledge Ms. Vanessa Andre for providing the drawing of pigs.

**Figure 2:**
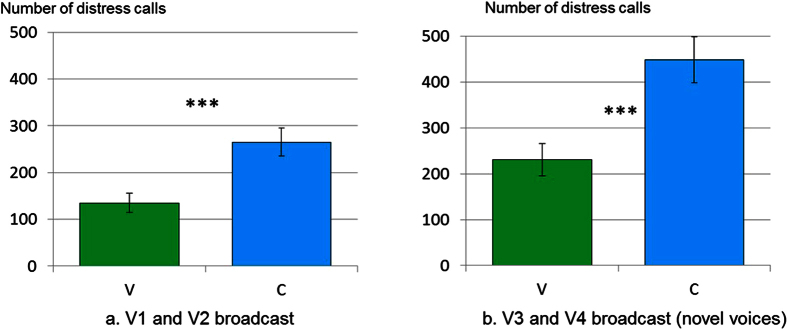
Mean (±sem) number of distress calls produced by piglets during isolation tests where human voices were broadcast according to their prenatal experience (V: prenatal experience of human voice: V1 and V2, N_a_ = 75 and N_b_ = 40; C: control without prenatal experience, N_a_ = 35 and N_b_ = 20). (**a**) Social isolation test at 2 days of age (SIT1) with broadcast of V1 and V2, and (**b**). social isolation test at 14 days of age (SIT3) with broadcast of V3 and V4 (novel human voices never heard by sows and piglets). Note that similar patterns are observed in the two situations with fewer distress calls in the experimental piglets when hearing any human voice but that this is more marked when the voices had been broadcast before birth.

**Figure 3:**
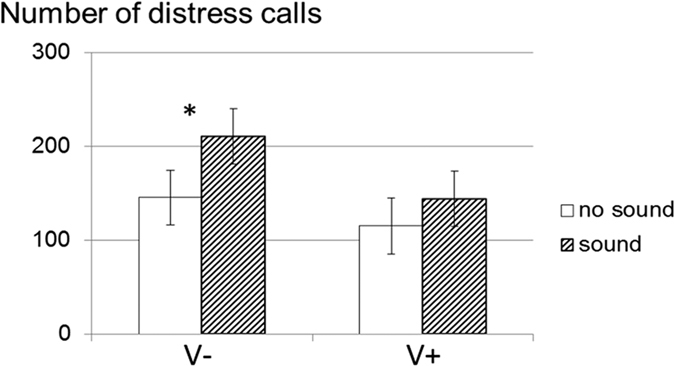
Mean (±sem) number of distress calls produced by 7 day-old piglets during Social Isolation Test 2 after prenatal experience (V: prenatal experience of human voice: V1 and V2, N_no sound_ = 33 and N_sound_ = 36): “No sound” - first five minutes of the test with no sound broadcast (Phase 1), “Sound” - last five minutes of the test with sound (V1 or V2) broadcast (Phase 2); “V-” the voice broadcast was associated with pregnant female negative handling (i.e. V2 for V1+ and V1 for V2+), “V+” the voice broadcast was associated with pregnant female positive handling (i.e. V1 for V1+ and V2 for V2+).

Three treatments were applied five days a week for the last month of gestation, gestation lasting in total 114 days (Fig. 1, Table 1, days -35 to -5 before birth). Treatments consisted of positive and negative handling sessions provided by two female experimenters (E1 and E2) with or without sound broadcast (V1 and V2 voices). Ten sows C received positive handling from E1 and negative handling from E2, without sound broadcast; ten sows V1+ also received positive handling from E1 and negative handling from E2, but with sound broadcasts (V1 voice during E1 handling and V2 voice during E2 handling); ten sows V2+ received the opposite, i.e. positive handling from E2 and negative handling from E1, with sound broadcasts. Sows from one treatment were reared in the same room without the possibility of hearing sounds from an adjoining room.

There were two 10 minutes sessions of human contact per day (×5 days per week, ×4 weeks): one of positive valence from one experimenter and one of negative valence from the other experimenter. One session took place in the morning and the other in the afternoon. Order of positive and negative sessions were counterbalanced over days resulting in an equal number of positive and negative handlings in the mornings and afternoons. Positive handling consisted of an alternation of sessions of brushing the back every 30 s and of spraying water in the mouth every 30 s. Negative handling comprised an alternation of sessions of abrupt gestures with a fly swatter toward the head every 30 s and of being touched on the hindquarters with an electric prod (ROBSET, 2500V) every 2.5 min with electric stimulations at 0 and 5 min.

Sows were kept in groups of six in 4 m × 7 m pens, on concrete floor with straw bedding till one week before farrowing. They were fed twice a day, before the treatment sessions. Then they were kept in individual farrowing pens (1.65 m × 2.40 m or 1.80 m × 2.65 m) till weaning. During handling sessions, sows were confined to individual feeding places so that the experimenter could face each sow during its handling session. The experimenter (regardless of treatment) stood less than 1 m from the sow and carried a loudspeaker hung around her neck facing the sow’s head. The experimenter stood and moved her arms to provide the treatment.

After each human action, we scored the sow’s reaction. The experimenter noted the presence/absence of a list of behavioural activities: grunts, distress vocalizations, postural changes, withdrawal, approach and aggressiveness. The data for the 20 sessions of each treatment were pooled for each individual. We calculated the percentage of actions in which each behaviour was observed.

### Piglets

In each litter, we aimed at testing four healthy, heavy piglets. This selection was made in order to decrease the risk of mortality that is quite high during the first days of life. We chose them after visual checking (no abscess, normal walking…). Each piglet was identified by an ear tag. Newborns were then submitted to different social isolation tests including playbacks according to Table 1. Due to the fact than some litters contained only small piglets, or when chosen piglets died before the test (poor quality of milk, digestive troubles…), we could not obtain the same sample size for each test. As a consequence, the number of piglets tested is specified for each test. The same person (MR) performed all experiments and analyses. Behaviours were sampled through direct observation preventing any blind scoring.

### Postnatal Social Isolation Tests (SIT) and playback experiments

At two days of age (SIT1), 110 piglets (35 “C” piglets, 36 “V1+”, and 39 “V2+”) were tested alone in a 1 × 2 m unfamiliar area where both V1 and V2 were being broadcasted. This procedure was repeated at 14 days of age (SIT3) with the broadcast of two unfamiliar voices (V3 and V4). Here, 20 other healthy piglets from each treatment were tested. These tests lasted for 5 minutes. The testing pen was made of plastic panels. Loudspeakers were positioned at each end and the sound level was fixed at 80 dB at 60 cm. Sounds were broadcast simultaneously. We alternated the source of the sounds between piglets in the same treatment, and the order of testing between the treatments and litters.

At 7 days of age (SIT2), piglets were tested in a 2 m × 2 m area for 10 minutes. During the first five minutes no sound was played back, and during the last five minutes recordings of a human voice were played back. For each treatment, half of the piglets were tested with V1 and the other half with V2 respectively (see sample sizes in Table 1). Piglets were chosen at random. Voices, treatments and litters were alternated on the test days.

Finally, at the age of 21 days (SIT4), piglets were placed in the presence of an unfamiliar female experimenter who remained motionless with the voice associated prenatally to mother’s positive handling. The test was done in the same pen as SIT2 and lasted for 5 min (see sample sizes in Table 1). Treatments and litters were alternated on the test days.

Our objective was to test the influence of the human voice on the level of stress experienced by piglets. Hence, for each test, we counted the total number of high-pitched distress calls emitted by each piglet. All piglets were included in the analysis.

### Statistics

To compare the behaviours of sows in the different situations, as data (i.e. proportions of occurrence of each behavioural reaction) were not distributed normally, we ran non-parametric tests. Statistics were done with StatView^®^ 5.0 (SAS Institute Inc, USA). The significance level was set at p < 0.05, and data in the text are presented as medians and quartiles. Mann-Whitney tests were used to compare the two independent groups constituted of the Experimental (V) and the Control (C) sows. Wilcoxon tests for dependent data were used to compare positive and negative handling sessions for the same individuals. In addition, Fisher’s exact tests allowed us to compare the number of sows expressing aggression between negative and positive sessions. To analyze the variations in piglets’ distress calling, we ran ANOVA Mixed Model analysis (NLME package in R) with mother and offspring identities as random factors. All meaningful factors were first included as fixed factors in the model (i.e. according to the experimental test: group, voice, voice emotional valence, phase - for SIT2 only). As there was no significant voice effect in any model tested (p > 0.05), data of V1+ and V2+ piglets were pooled and then called group V. To control for litter effects, the litter was added as a random effect in all models. We thus compared V and C piglets in all situations and we compared Phase 1 and Phase 2 in SIT2 situation.

## Additional Information

**How to cite this article**: Tallet, C. *et al*. Postnatal auditory preferences in piglets differ according to maternal emotional experience with the same sounds during gestation. *Sci. Rep*. **6**, 37238; doi: 10.1038/srep37238 (2016).

**Publisher’s note**: Springer Nature remains neutral with regard to jurisdictional claims in published maps and institutional affiliations.

## Figures and Tables

**Table 1:** Timeline of the experiment.

Day	Who	Event	Observation	Place	Sample size		
					C	V1+	V2+
PND-30 to PND-5	Sows	Positive and negative handling treatments		Rearing pen	10	10	10
D0	Sows	Delivery		Rearing pen	9	10	9
D2	Piglets	Social Isolation Test 1 (SIT1) (5 min)	Simultaneous broadcast of V1 and V2 voices	Testing pen 1 × 2 m	35	36	39
D7	Piglets	Social Isolation Test 2 (SIT2) (10 min)	Piglet alone during two consecutive phases of 5 minutes each: Phase 1: No sound broadcast Phase 2: Sound broadcast (either V1 or V2)	Testing pen 2 × 2 m	34 (V1: 17, V2: 17)	36 (V1: 18, V2: 18)	37 (V1: 19, V2: 18)
D14	Piglets	Social Isolation Test 3 (SIT3) (5 min)	Simultaneous broadcast of V3 and V4 voices	Testing pen 1 × 2 m	20	20	20
D21	Piglets	Social Isolation Test 4 (SIT4) (5 min)	Piglet with a motionless human and broadcast of the voice associated prenatally with the maternal positive treatment (i.e. V1 for V1+ and V2 for V2+)	Testing pen 2 × 2 m	16	18	18
